# Insulin-Like Growth Factor Pathway and the Thyroid

**DOI:** 10.3389/fendo.2021.653627

**Published:** 2021-06-04

**Authors:** Terry J. Smith

**Affiliations:** Department of Ophthalmology and Visual Sciences, Kellogg Eye Center, Division of Metabolism, Endocrinology, and Diabetes, Department of Internal Medicine, University of Michigan Medical School, Ann Arbor, MI, United States

**Keywords:** growth factor, hormone, goiter, autoimmune, Graves’ disease, ophthalmopathy, thyroid

## Abstract

The insulin-like growth factor (IGF) pathway comprises two activating ligands (IGF-I and IGF-II), two cell-surface receptors (IGF-IR and IGF-IIR), six IGF binding proteins (IGFBP) and nine IGFBP related proteins. IGF-I and the IGF-IR share substantial structural and functional similarities to those of insulin and its receptor. IGF-I plays important regulatory roles in the development, growth, and function of many human tissues. Its pathway intersects with those mediating the actions of many cytokines, growth factors and hormones. Among these, IGFs impact the thyroid and the hormones that it generates. Further, thyroid hormones and thyrotropin (TSH) can influence the biological effects of growth hormone and IGF-I on target tissues. The consequences of this two-way interplay can be far-reaching on many metabolic and immunologic processes. Specifically, IGF-I supports normal function, volume and hormone synthesis of the thyroid gland. Some of these effects are mediated through enhancement of sensitivity to the actions of TSH while others may be independent of pituitary function. IGF-I also participates in pathological conditions of the thyroid, including benign enlargement and tumorigenesis, such as those occurring in acromegaly. With regard to Graves’ disease (GD) and the periocular process frequently associated with it, namely thyroid-associated ophthalmopathy (TAO), IGF-IR has been found overexpressed in orbital connective tissues, T and B cells in GD and TAO. Autoantibodies of the IgG class are generated in patients with GD that bind to IGF-IR and initiate the signaling from the TSHR/IGF-IR physical and functional protein complex. Further, inhibition of IGF-IR with monoclonal antibody inhibitors can attenuate signaling from either TSHR or IGF-IR. Based on those findings, the development of teprotumumab, a β-arrestin biased agonist as a therapeutic has resulted in the first medication approved by the US FDA for the treatment of TAO. Teprotumumab is now in wide clinical use in North America.

## Introduction

Among the most ubiquitous regulatory factors governing functions in the mammalian body are those belonging to the insulin and insulin-like growth factor-I (IGF-I) family, including their respective receptors, binding and related proteins, and extensive signaling pathways that mediate/modulate their actions. Discovery of insulin has been attributed to several individuals, but most prominent among them are Paulescu [cited in reference (1)] in France and Banting, Best and colleagues (2) in Toronto, Canada, working independently. Therapeutic insulin was first administered to patients with diabetes mellitus in 1922, representing a seismic event ranking among the most impactful in modern medicine. In aggregate, this body of work concerning the discovery and characterization of insulin opened the door to our current understanding of normal and pathological carbohydrate metabolism with implications extending far beyond. Its “first cousin”, sulfation factor, was first described nearly four decades later by Daughaday and colleagues (3). Sulfation factor underwent a name change to somatomedin C in 1972 (4) and finally to its current designation, IGF-I, in the early 1980s (5). Subsequent to their discovery, the actions of both insulin and IGF-I have been characterized extensively and found to be overlapping in many regards. Their physiological roles and impact on target tissues and cells have been slowly disentangled but their functional promiscuity and that of their respective receptors continue to intrigue students of the field.

In this article, I focused on the growth hormone (GH) and IGF-I pathways and have attempted to briefly review their numerous intersections in the hypothalamic-pituitary-thyroid axis. The relevance of IGF-IR in the pathogenesis of thyroid-associated ophthalmopathy (TAO), the most serious extra-thyroidal autoimmune manifestation of Graves’ disease (GD), has only come into prominence over the past 2 decades (6–8). As I will describe, IGF-IR plays not only a critical role in the pathogenesis of TAO, but can be effectively targeted as a therapeutic strategy for managing the disease. The concept of IGF-IR playing an important role in the development of TAO or targeting the protein as a strategy for treating the disease had been met with overwhelming skepticism when it was first proposed (9, 10). Despite these dismissive views, teprotumumab, an inhibitory monoclonal antibody directed at IGF-IR, was recently approved specifically for the treatment of TAO. It is now in clinical use in North America; however, considerably more information will be required if we are to fully understand its mechanism of action in ameliorating the signs and symptoms of TAO and the entirety of its off-target consequences on the human body.

### IGF-I and Its Associated Pathway

**Figure 1:**
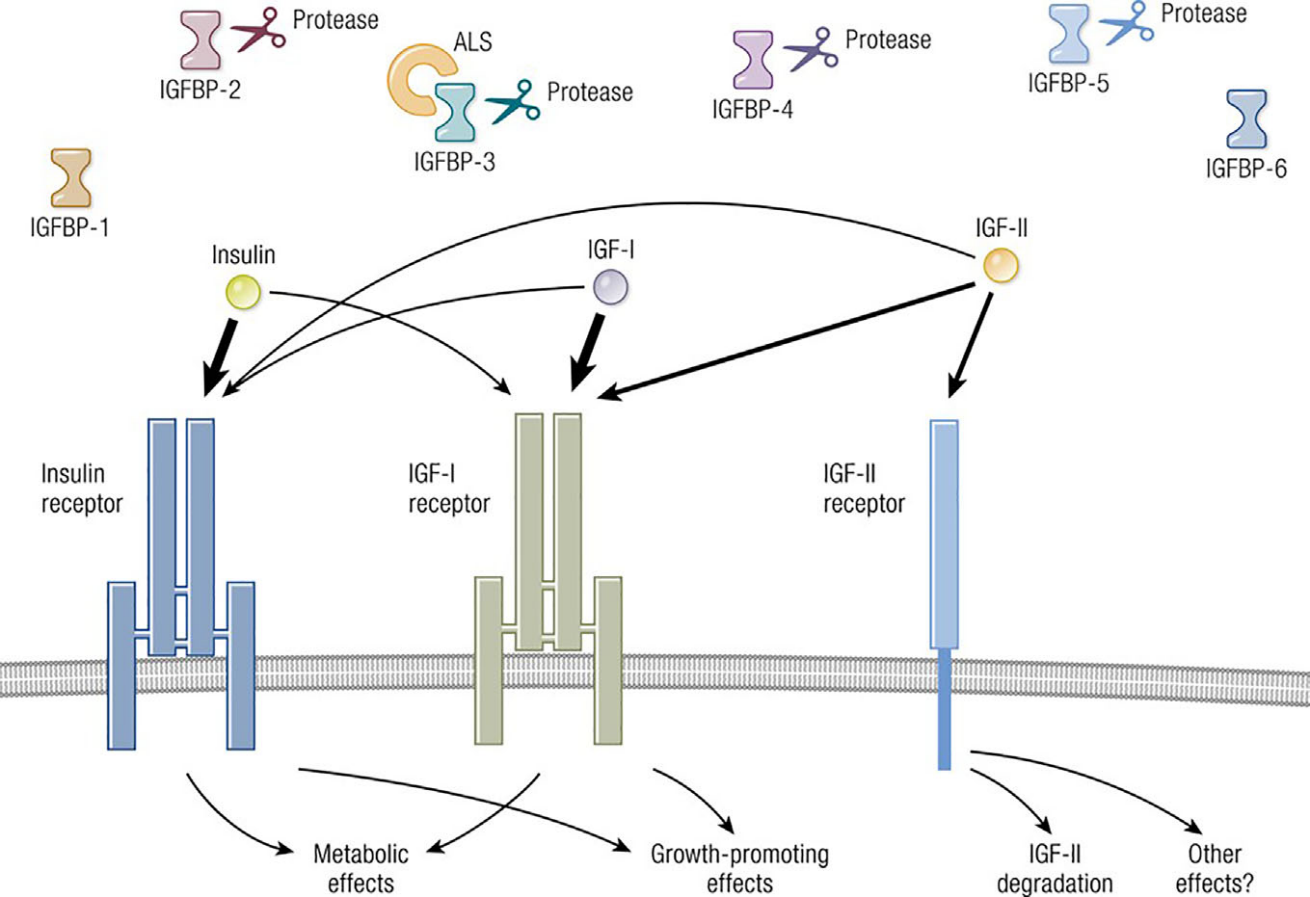
Overview of the IGF-I pathway, including its multiple ligands, modulatory binding proteins and receptors. Ligands including IGF-I, IGF-II, and insulin share important structural and functional relationships including the promiscuous utilization of multiple receptors, such as IGF-IR, IGF-IIR, and IR. The pathway also contains IGF-binding proteins (IGFBPs) which can function either independently or may require ligation. IGF-IR plays a central role in mediating growth regulation. In contrast, the insulin receptor is the primary regulator of metabolism and carbohydrate handling. IGF-IIR (mannose-6-phosphate receptor) influences IGF-II degradation and participates in the biology of the Golgi apparatus. Biological impact of both IGF-I and IGF-II action is modulated by six thus-far identified IGFBPs. Adapted from Lowe WL. Insulin-like growth factors. Science & Medicine 1996; 3; 65.

**Figure 2:**
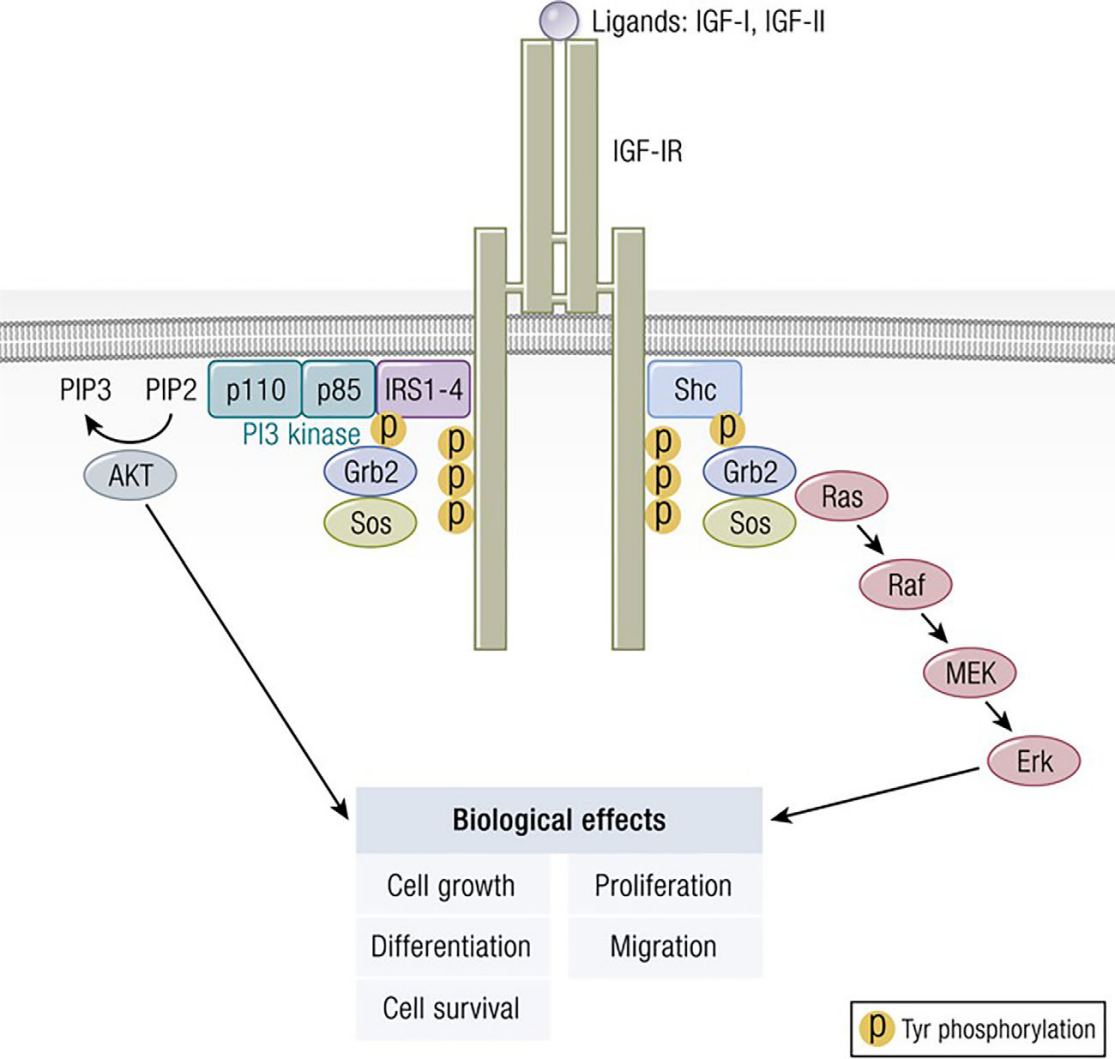
Classical model of signaling through the IGF-IR. IGF-I ligation of IGF-IR most commonly results in canonical mitogen activated protein kinase (MAPK)/Ras-Raf-Erk and phosphatidylinositol-3-kinase/AKT/mTOR (PI3K/AKT) phosphorylation, critical to downstream signaling and the activation of targeted genes associated with IGF-I responses. Adapted from Worrall et al. Novel Mechanisms of Regulation of IGF-1R Action: Functional and Therapeutic Implications. Pediatr Endocrinol Rev. 2013 10:473-484.

The molecular structure of IGF-I is closely related to insulin (11) (**Figure 1**). IGF-I comprises 70 amino acids and mediates growth during childhood and adolescence (12). IGF-II contains 67 amino acids. Both IGF-I and IGF-II have three intramolecular disulfide bridges. While IGF-I synthesis was initially considered to be driven by GH as a systemically active mediator, recent decades have witnessed increasing insight into its roles as a paracrine and autocrine factor. Mediating the actions of insulin and IGF-I are their respective, closely related receptors (IGF-IR and IR, respectively), both belonging to the tyrosine kinase family (**Figure 2**). The cell surface receptors of both ligands also exhibit substantial structural and functional relatedness and may have evolved from gene duplication of a common precursor (13). They form IR/IR and IGF-IR/IGF-IR as well as IR/IGF-IR hybrids, the particular protein pairings of which might be determined stochastically. Both possess heterotetrameric protein structures comprising extracellular domains containing ligand binding sites located in two α subunits. Two β subunits consist of extracellular, transmembrane and intracellular domains containing the kinase domains, the ATP binding site, and multiple potential tyrosine phosphorylation sites (14). The α and β subunits are linked by two disulfide bonds. Both insulin and IGF-I engage in promiscuous interactions with each other’s receptor, causing substantial overlap in the physiological and pathological consequences of activating the IGF-IR and IR pathways. While IGF-IR primarily functions as an integral membrane receptor the activation of which provokes tyrosine kinase autophosphorylation, recent studies have revealed the nuclear translocation of IGF-IR as well as other components of the IGF-I pathway (15). Similar to many other cell-surface receptors, the density of IGF-IR determines in part the pattern of signaling downstream from that receptor (16). It has been proposed that unligated IGF-IR can initiate signaling mediated through microRNAs and imprinted genes (17). Several factors can upregulate IGF-IR gene expression while others reduce target gene transcriptional activity (8). In addition to IGF-IR, a second receptor, IGF-IIR, aka the mannose-6-phosphate receptor, can bind IGF-II (18) and plays roles in trafficking molecules to the Golgi and the endosomal-lysosomal system (19). IGF-IIR does not mediate tyrosine phosphorylation-dependent signaling analogous to that attributed to IGF-IR and IR. Besides the two receptors, the IGF-I pathway includes six IGF-I binding proteins and nine IGFBP related proteins (20, 21). Their potential relevance to the thyroid has been reviewed recently in detail (8).

## Impact of GH and its Primary Effector, IGF-I, on the Thyroid Gland

The relationship between GH, IGF-I and the thyroid gland is complex, frequently reciprocal and temporally spans the interval during fetal development throughout adult life (22, 23). Much of what we understand about the impact of the IGF-I pathway on the thyroid derives from observations made in states of hormone excess such as acromegaly (22, 24). Porcine thyroid cells express both IGF-IR and IR (25) as do human thyroid cancer cells and normal thyroid (26). Thyrospheres derived from healthy tissue express both isoforms of IR, namely IR-A and IR-B, as well as IGF-IR, IGF-I and IGF-II, levels which decline following cell differentiation. Interestingly, between the two insulin receptor isoforms, IR-A expression dominates thyrospheres while IR-B is more abundant in differentiated thyrocytes. IGF-I binding is saturable and Scatchard analysis discloses a single binding class with a Ka 4.3 X 10^-10^ and 49 X 10^3^ sites per cell. IGF-I stimulates DNA synthesis and thyroid cell proliferation, actions synergistic with those of epidermal growth factor. IGF-I and insulin have important roles in regulating not only proliferation but also thyroid cell differentiation. In studies of FRTL-5 rat thyroid cells, IGF-II but not IGF-I was found to be synthesized and released (27). IGF-II promotes DNA synthesis in FRTL-5. On the other hand, medullary thyroid carcinoma cells were found to express both IGF-I and IGF-II (28, 29). A p21 Ras mutation can induce IGF-I expression and release in immortalized human thyroid epithelial cells (30). In porcine thyroid follicles, TSH and iodine were found to regulate IGF-I mRNA levels (31). In those studies, IGF-I transcript was undetectable in the absence of TSH, effects that were mimicked by forskolin. In contrast, Iodine downregulated the levels of this mRNA. Similar TSH-dependent effects on IGF-I were demonstrated in another report (32). Interestingly, differentiation of mouse embryonic stem cells to thyroid epithelial cells requires insulin/IGF-I, suggesting the complexities of these factors in thyroid development (33). The incidence of thyroid nodules was found to be increased substantially in children with extreme insulin resistance (34) bringing clinical relevance to the basic observations made in the laboratory.

Signaling through both IGF-IR and IR is considered essentially indispensable in the normal function of the thyroid gland, both through their independent support of thyroid epithelial survival and vitality and also by virtue of the interactions of their downstream pathways with those of the thyrotropin receptor (TSHR) (35). The patterns of effects in FRTL-5 cells suggest that IGF-I and insulin have distinct actions on specific gene expression, both independent of and in concert with TSH (36). Among the IGF-IR/IR-activated pathways in thyroid cells are Erk 1/2, and Akt, while TSHR is G protein coupled and results in the generation of cAMP. Examination of IGF-IR and insulin receptor docking proteins has revealed a role for insulin receptor substrate 2 (IRS-2) in mediating the proliferative actions of IGF-I, both *in vitro* and *in vivo* (37). Iodide uptake and organification are among the specific aspects of thyroid metabolism under dual TSH and IGF-I/insulin control. These processes in turn are mediated through the activities of the sodium iodide symporter and thyroperoxidase, respectively. In a study of mice with conditional double-thyroid knockout of IGF-IR and IR, neonatal thyroid glands were smaller, exhibited repressed FOXE1 expression, and manifested defective folliculogenesis (38). At postnatal day 14, mTOR-dependent epithelial cell proliferation and serum TSH were detectable. By week 50, lesions resembling papillary carcinoma had developed, coincident with ErbB activation. The authors concluded that both IGF-IR and IR are critical to follicle formation in the developing thyroid. IGF-IR engagement in cultured thyroid epithelial cells has been shown to activate the expression of chemokines and cytokines such as IL-16 and RANTES (39). Both insulin and IGF-I can regulate the rate of major histocompatibility complex class 1 (MHC class 1) gene transcription (40). These effects are similar to those of TSH in FRTL-5 cells. IGF-I and insulin in combination with TSH can suppress the transcription of the Mac-2BP gene in these cells by decreasing the binding of an upstream specific factor to a gene promoter site (41). IGF-I and cAMP appear to differentially activate the PI3 kinase pathway in FRTL-5 cells, leading to G1 cyclin-cyclin dependent kinase activation and DNA synthesis (42). IGF-I induces several anti-apoptotic proteins in thyroid cells, including Fas-associated death domain-like interleukin-1-converting enzyme-inhibitory protein, actions mediated through activation of the NF-κB pathway (43). Quercetin inhibition of FRTL-5 proliferation is mediated by alterations in insulin and IGF-I signaling involving the Akt pathway (41). It has been suggested that lithium might influence IGF-IR coupled G_i_-proteins during the G1 phase of FRTL-5 cell cycling (44). With regard to thyroid cancer, the IGF-I pathway has been directly implicated in the transformation of thyroid epithelial cells (45). It remains uncertain whether the relationship between IGF-I action, IGF-IR signaling, or any of the other components of the IGF-I pathway differ in their relationship to thyroid cancers when compared to other forms of neoplastic disease. Elevated levels of the IGF-IEc isoform have been reported in differentiated papillary thyroid carcinoma and are associated with advanced disease (46).

## The Thyroid in Acromegaly

The hypothalamic-pituitary-thyroid axis can be affected at several levels in acromegaly, including those imposed directly and indirectly on the gland itself. For instance, interpretation of laboratory thyroid testing in this condition can be complicated. Both basal and pulsatile TSH secretion are often reduced while thyroxine levels can remain unaffected (47). This phenomenon may result from enhanced thyroid sensitivity to the actions of TSH caused by either growth hormone, IGF-I, or both. In their very recent report, Natchev and colleagues studied a cross-section of 146 acromegalic patients and found that secondary hypothyroidism and hyperthyroidism are commonly encountered (48). Thyroid gland volume is frequently impacted (49) and frank thyroid enlargement in acromegaly is not unusual. Thyromegaly was first recognized in acromegaly by Rolleston in 1936 (24). Gland volume in acromegaly correlates with serum GH and IGF-I levels. Distinguishing which of these hormones might be impacting thyroid enlargement in a particular case is frequently not possible (50). The incidence of both euthyroid and hyperthyroid goiters is increased in acromegaly, a consequence potentially independent of TSH (51). In the absence of TSH, it appears that GH has little or no effect on thyroid size (52). Surgical cure of acromegaly can result in reduced thyroid volume (50). Sera from patients manifesting untreated acromegaly can induce [^3^H] thymidine incorporation into the DNA of FRTL-5 cells more than sera from healthy control donors (53). The mitogenic activities of both normal and acromegalic sera can be attenuated by pretreating them with neutralizing monoclonal anti-IGF-I antibodies. Thyroid enlargement in acromegaly can be nodular, multinodular or diffuse (48, 54). The nodules in this condition are described as “stiff”, a quality thought to result from localized fibrosis (55). Besides benign goiter, the incidence of thyroid carcinoma is said to be increased in acromegaly (56, 57). In fact, both benign and malignant thyroid nodules are commonplace in these patients (54). Patients with acromegaly are at increased risk for a variety of cancers besides those of the thyroid. These include those of the colon, prostate, and breast, but thyroid cancers may be the most common malignancy associated with the disease (58). IGF-I can promote tumor progression and perhaps facilitate neoplastic initiation (59). McCune-Albright syndrome occurring in adults can be associated with toxic multinodular goiter and an increased risk of thyroid cancer (60). Of the thyroid cancers associated with acromegaly, papillary tumors can occur, especially in those patients manifesting other endocrine and non-endocrine neoplasms. These include pheochromocytoma, growth hormone-producing pituitary adenoma, and duodenal adenocarcinoma (61, 62).

## Interplay Between IGF-I and Thyroid Hormone Metabolism and Pathways

As suggested above, entanglement between the actions of GH, IGF-I and thyroid hormones is complex and can become unmasked with excesses or deficiencies in one or both pathways. Childhood deficiency of GH can occur as an isolated defect, whereas those occurring in adults are typically associated with a constellation of pituitary-hypothalamic insufficiencies and are mostly associated with anatomic lesions. Exogenous GH administration in GH-deficient children has been shown to reduce circulating levels of T_4,_ although the mechanism(s) involved has yet to be established. Some studies have revealed a GH-dependent increase in T_4_ to T_3_ conversion (63) and that GH may support physiological T_4_ to T_3_ conversion. Further, GH deficiency may impair T_3_ generation (64). GH may also work centrally, either through direct actions or those mediated through IGF-I to reduce the synthesis and release of TSH from the pituitary (65). T_3_ and IGF-I influence divergently GH synthesis and release from cultured pituitary cells in monolayer (66). The enhancement of GH synthesis by T_3_ is mediated through enhanced gene transcription while the inhibition by IGF-I of GH induction appears to reflect changes in posttranscriptional events. Insulin and thyroid hormones can act synergistically by mutual enhancement. For instance, T_3_ can upregulate the expression of glutamic acid decarboxylase in pure cortical neuronal cultures, actions requiring the presence of insulin (67). T_3_ (and thyroxine to a lesser extent) enhances the increased sulfation of embryonic cartilage by sera from either intact or hypophysectomized rats, effects mediated at least in part by IGF-I (68). IGF-I and thyroid hormones interact on growth plate chondrocytes where IGF-I enhances Wnt-4 expression and β-catenin activation (69). In that study, T_3_ was shown to enhance IGF-1R signaling and its upregulation of PI3K/Akt/GSK-3β signaling. IGF-I increases levels of thyroid transcription factor-2, actions similar to those of insulin (70). T_3_ and IGF-I have synergistic, enhancing effects on the expression of fast-type sarcoplasmic-reticulum Ca^++^-ATPase in L6 myocytes (71). T_3_ acts on the rate of gene transcription while IGF-I enhances mRNA and protein stabilities. These interactions can translate into clinically relevant events. For instance, exogenous GH attenuates thyroid hormone action in patients with Turner syndrome (72).

## Impact of Thyroidal Status on the GH/IGF-I Axis

Normal growth requires intactness of both GH/IGF-I and thyroid hormone pathways. Thyroid hormones influence IGF-I levels in the pituitary and in peripheral tissue compartments (73), including expression and release of GH from the pituitary (74, 75) and the circulating levels of both GH and IGF-I (76). Animals harboring the mutant transcription factor, Pit-1, (Snell dwarf mice), exhibit multiple deficiencies in anterior pituitary hormones resulting in defective B cell development (77). This defect can be partially ameliorated with exogenous GH or IGF-I but a more complete remediation can be accomplished with administration of exogenous thyroxine. Thus, the regulatory actions of GH/IGF-I and TSH/thyroid hormones appear to overlap in bone marrow cells. Evidence suggests that not all thyroid hormone effects on the IGF-I pathway are mediated through GH expression and release but that other, GH-independent mechanisms may also be involved (78). Some contradictory findings have been reported in human dysthyroidemia. Plasma levels of IGF-I were found to be reduced in hypothyroid individuals; these were increased substantially with adequate thyroid hormone replacement. Basal IGF-I levels are lower in hypothyroid patients compared to those who are euthyroid (79). In another report, serum IGF-I levels were lower in hyperthyroid patients while levels trended toward elevation in hypothyroid individuals (80). Other components of the IGF-I pathway are influenced by thyroid hormone. Thyroxine replacement therapy appears to increase serum levels of IGFBP1 (81). In 18 day old rats, thyroid hormones can regulate the expression of hepatic IGFBP2 mRNA and serum protein levels through a mechanism independent of GH (78). Thus thyroid hormone effects on IGFBP2 in these animals diverge from those of IGF-I where its effects are mediated through GH. Serum IGFBP3 and IGFBP4 levels are reduced in hypothyroid animals (82). Thyroidectomized patients in whom thyroid hormone replacement has been withdrawn exhibit a reduction in circulating IGFBP1 levels (83) while thyroxine treatment increases them (81). It appears that the impact of thyroid hormone status on the GH/IGF pathway may change with maturation; further some of these developmental-stage sensitive effects are mediated through GH (78). Propylthiouracil treatment in rats results in substantially increased IGF-II binding site density on thyroid epithelial cells (84). In contrast, thyroid hormones induce IGF-IR mRNA expression in rat epiphyseal chondrocytes (85) and enhance IGF-I binding in rat pituitary cells (86). They regulate IGF-IR expression in rat heart and lung, both during animal development and in adults (87). In aggregate, these findings suggest the complex interactions shared by the two pathways. They support the important roles played by each in the maintenance of both endocrine functions at multiple levels.

## IGF-I and Insulin Enhance the Actions of TSH and Thyroid-Stimulating Immunoglobulins: Evidence for Interplaying Pathways

**Figure 3:**
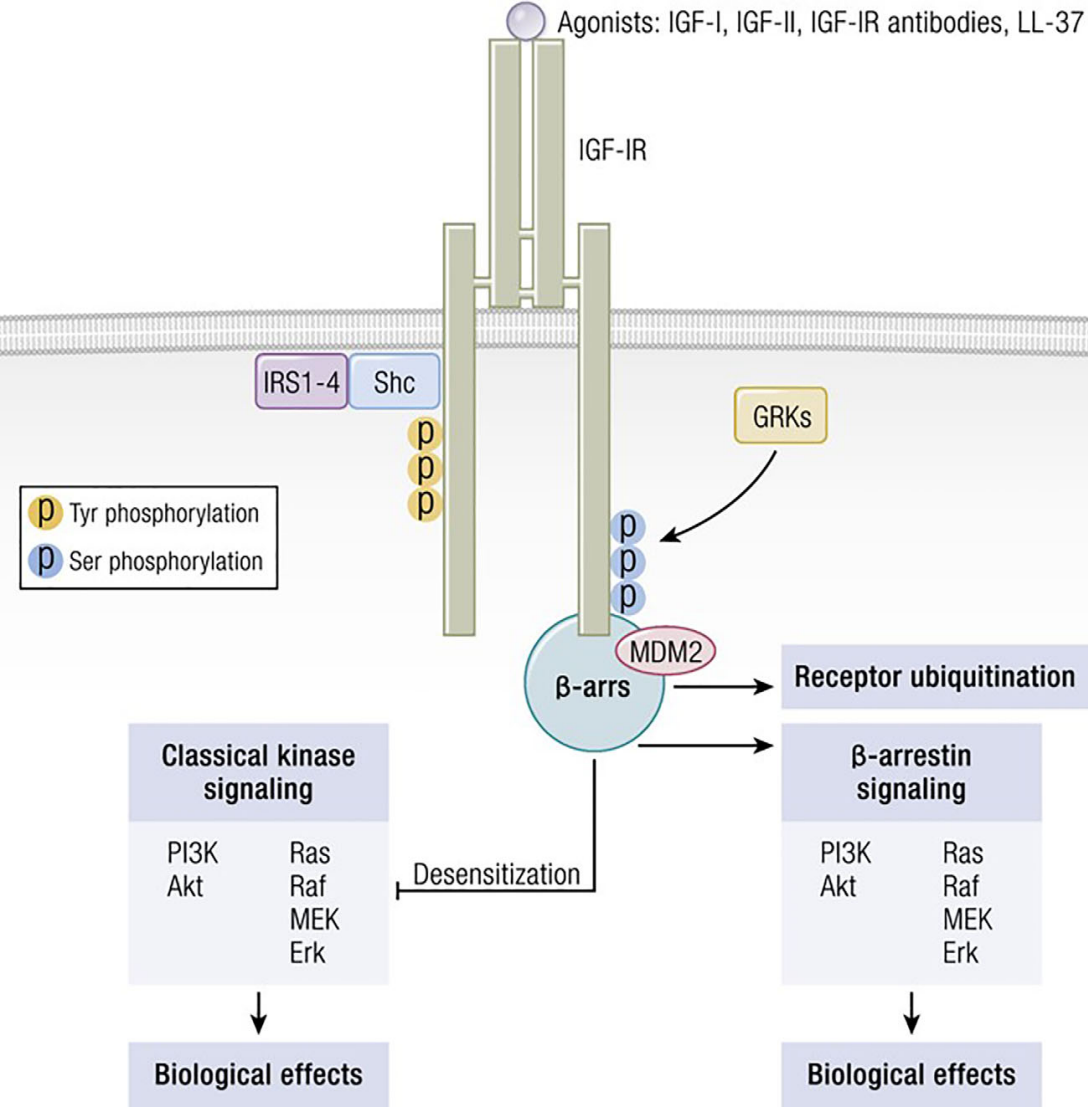
IGF-IR can form functional RTK/GPCR hybrids, thus aggregating tyrosine kinase and GPCR signaling. Ligand-dependent activation of classical kinase-dependent signaling with β-arrestin recruitment provokes GRK-dependent serine phosphorylation located in the IGF-IR C-domain. β-arrestin activates kinase desensitization and ubiquitination and initiates kinase-independent signaling through MAPK. Adapted from Worrall et al. Novel Mechanisms of Regulation of IGF-1R Action: Functional and Therapeutic Implications. Pediatr Endocrinol Rev. 2013 10:473-484.

Several factors are necessary for thyroid epithelial cells to function normally. Among these are IGF-I and TSH, both of which are critical to thyroid hormone synthesis (88). Of these two molecules, TSH and its cognate receptor, TSHR, represent the primary regulatory pathway for thyroid development, growth and function. Several ‘well-travelled” IGF-IR signaling pathways cross-talk with those downstream from TSHR in thyroid epithelial cells. These interactions can either enhance or modulate TSHR-mediated biosynthetic events, depending on their context. Among the intersecting signaling cascades, the p42/44 mitogen-activated protein kinase (MAPK) pathway is pivotal (89). TSHR signaling through p42/44 MAPK is independent of cAMP but dependent on the receptor’s coupling to G13 protein (89). So too, is this pathway of central importance to the signaling initiated through IGF-I. The interplay between the actions of IGF-I, insulin and TSH in the thyroid involves interactions between tyrosine kinase receptors (RTKs) and G protein coupled receptors (GPCRs) (**Figure 3**). These receptor classes share scaffolding proteins, namely β-arrestin 1 and 2. The apparently critical role played by β-arrestin 1 in downregulating IGF-IR expression through ubiquination is mediated by an MDM2 E3 Ligase dependent mechanism (90). β-arrestin also mediates IGF-IR signaling through the Erk pathway (91). Studies in Ewing’s sarcoma cells treated with figitumumab, an IGF-IR antagonist, revealed that this antibody functioned as an IGF-1R-biased agonist through β-arrestin1 recruitment to the receptor, enhancing IGF-IR ubiquination and provoking Erk phosphorylation in the absence of Akt activation (92). The scope of potential RTK/GPCR hybrid formation is not limited to that involving the IGF-I/insulin family. These hybrids have been observed with other RTKs, including the epidermal growth factor receptor (93). Initial observations that IGF-I and insulin can amplify effects of TSH on thyroid epithelial cells were conducted in FRTL-5 cells (94). Ingbar and colleagues found that both IGF-IR- and IR- activating ligands could provoke proliferation and DNA synthesis in these cultured cells. Their initial report was soon followed by more complete characterizations from that group as well as studies emanating from other laboratories (95). Those studies disclosed that IGF-I could consistently enhance TSH actions in thyroid epithelial cells and demonstrated synergism with regard to cell proliferation and tyrosine kinase activation. As an example, TSH and IGF-I synergistically enhance levels of 1,2-diacylglycerol in FRTL-5 cells, resulting in increased DNA synthesis (96). On the other hand, TSH and IGF-I have distinctly different effects on immediate early gene expression in rat thyroid epithelial cells (97). Thus, despite similarities, the two pathways should be viewed as non-equivalent but frequently intersecting. Tsui et al. subsequently reported that signaling initiated by both rhTSH and GD-IgG could be attenuated *in vitro* by 1H7, a monoclonal anti-IGF-IR-inhibitory antibody (98). Those investigators also reported that TSHR and IGF-IR co-localize in orbital fibroblasts, *in situ* in orbital fat and in primary human thyroid epithelial cells. Further, the two receptor proteins co-precipitate in monoclonal antibody-utilizing pull-down studies (98). Those studies were followed by others demonstrating that the conditional knock-out of IGF-IR in thyroid diminishes its responsiveness to TSH (99). Conversely, selective, combined over-expression of IGF-I and IGF-IR in thyroid of transgenic mice exhibits enhanced sensitivity to endogenous TSH (100). Extensive crosstalk of the multiple downstream signal transduction pathways utilized by the two receptors has been identified (101–104). Those pathways include Erk 1/2 (98). Further, TSH can enhance IGF-I signaling in thyroid, actions mediated through the generation of cAMP (105). Thus, there exists substantial molecular rationale for considering the importance of a functional interplay between IGF-IR and TSHR and for targeting this protein complex therapeutically. Congruent with that possibility, Chen et al. (106) found that the fully human IGF-IR inhibiting antibody, teprotumumab, examined earlier in clinical trials for multiple cancers (107–111), could also attenuate the actions of both IGF-I and TSH in cultured CD34^+^ fibrocytes. IGF-I was recently found to enhance the expression of TSHR in orbital fibroblasts (112).

## Emerging Relevance of IGF-IR in the Pathogenesis of GD and TAO

**Figure 4:**
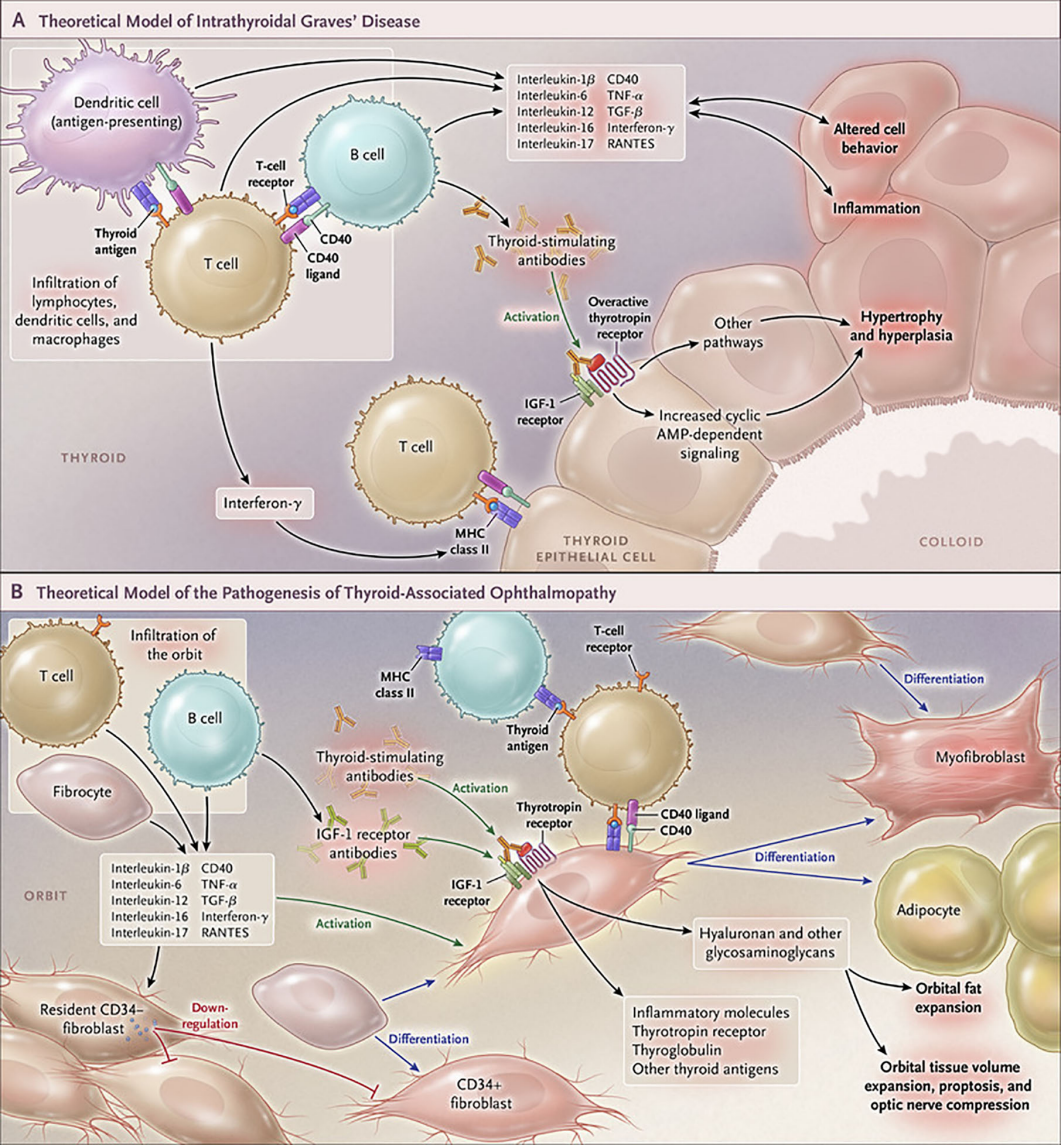
Cartoon proposed model of Graves’ disease and thyroid-associated ophthalmopathy (TAO) pathogenesis. **(A)** Thyroid-stimulating immunoglobulins (TSI) drive the over-production of thyroid hormones by activating the thyrotropin receptor (TSHR), thus overriding the normal regulatory role of thyrotropin on the process. B and T cells and antigen-presenting cells which infiltrate the gland produce interleukins 1β, 6, 12, 13, interferon γ, tumor necrosis factor α, and CD40 ligand. These cytokines then activate thyroid epithelial cells, promote inflammation, and induce genes ordinarily unexpressed by these cells such as major histocompatibility complex II (MHC II). Anti-thyroid drugs are useful therapeutically by reducing excess thyroid hormone production as well as modulating the expression of pathogenic intrathyroidal cytokines. **(B)** The pathogenesis of TAO also involves the infiltration of professional immune cells. Orbital fibroblasts exhibit particularly robust responses to inflammatory mediators. Among these cells are CD34^+^ fibroblasts which we propose derive from fibrocytes, monocyte-derived progenitor cells that traffic from bone marrow. Fibrocytes circulate in Graves’ disease at higher frequency than that found in healthy individuals. When cultivated from the peripheral circulation, fibrocytes express several thyroid-specific proteins, including thyrotropin receptor (TSHR), thyroglobulin, thyroperoxidase and sodium-iodide symporter. They also express MHC constitutively and can present antigens. When exposed to the appropriate culture conditions, they undergo differentiation into myofibroblasts (through Smad pathway activation by TGF-β) and adipocytes (through the activation of PPAR-γ). Many of the genes expressed by fibrocytes are detected at considerably lower levels in CD34^+^ orbital fibroblasts. We have found recently that these lower levels of expression result from the actions of Slit2 which acts through its cognate receptor, Roundabout 1 (ROBO1). When activated, CD34^+^ fibrocytes and CD34^+^ fibroblasts generate several pro-inflammatory or anti-inflammatory cytokines, including interleukins 1β, 6, 8, 10, 12, 16, tumor necrosis factor α, and regulated on activation, normal T expressed and secreted (RANTES), CXCL-12 and CD40-CD154. Both CD34^+^ and CD34^-^ orbital fibroblasts cell-surface display insulin-like growth factor-I receptor (IGF-IR). Orbital fibroblasts express three mammalian hyaluronan synthase (HAS) isoenzymes and UDP glucose dehydrogenase and synthesize hyaluronan, the glycosaminoglycan associated with expanding orbital tissue in TAO. The vast majority of hyaluronan synthesis occurs in CD34^-^ orbital fibroblasts. From N. Engl. J. Med, Smith T.J. and Hegedus L., Graves’ Disease, 375; 1552-1565. Copyright ^©^ (2016) Massachusetts Medical Society. Reprinted with permission.

The development of GD remains an only partially understood process. These deficits have resulted historically in suboptimal medical management of both the thyroid dysfunction and TAO components of this vexing condition (113) (**Figure 4**). Initial insights that the IGF-I pathway might be involved in the pathogenesis of TAO emerged from the study of Weightman et al. (114). These investigators reported detecting IgGs collected from patients with GD (GD-IgG) that were capable of displacing radiolabeled IGF-I from binding sites on the surface of orbital fibroblasts coming from these individuals (GD-OF). Pritchard et al. demonstrated subsequently that these bindings sites were IGF-IR rather than one or more of the IGFBPs (9). Further, Pritchard et al. reported that GD-IgGs can initiate signaling in GD-OF, resulting in the activation of the PI_3_ kinase/FRAP/mTOR/p70^s6 kinase^ pathway and the induction of target genes encoding IL-16 and “Regulated Upon Activation, Normal T Cell Expressed and Presumably Secreted” (RANTES), two T cell chemoattractants (115). A major issue remaining to be clarified concerns whether the agonistic autoantibodies generated in TAO that induce responses in GD-OF and fibrocytes act directly through TSHR, IGF-IR or both. Whether the anti-IGF-IR antibodies represent those that stimulate or block the receptor’s activation or do neither (neutral) remains another open question.

IGF-IR is over-expressed by GD-OF (9) as well as T cells (116) and B cells (117). This increased IGF-IR expression in patients with GD is undetectable in the unaffected monozygotic twin of a sibling with GD, strongly suggesting that non-genetic factors are responsible, at least in part, for the increased receptor levels associated with the disease (118). IGF-I and GD-IgG purified from patients with GD were found to enhance hyaluronan accumulation in cultured GD-OF but had no effect in orbital fibroblasts from healthy donors (119). In contrast, rhTSH failed to influence glycosaminoglycan synthesis. Further, IGF-I appears to skew the accumulating HA molecules toward higher molecular weight species and to promote the proliferation of perimysial GD-OF (120). The effects of hyaluronan on proliferation of these fibroblasts was found to involve differential effects on membrane polarization, where high molecular weight hyaluronan results in depolarization and low molecular weight hyaluronan causes membrane hyperpolarization and inhibits proliferation.

As mentioned above, subsequent studies have disclosed the physical and functional interactions between IGF-IR and TSHR occurring in thyroid epithelial cells, GD-OF and *in situ* in TAO orbital fat (98). Those studies of Tsui et al. also demonstrated that inhibiting IGF-IR could attenuate Erk 1/2 p42/44 activation, regardless of whether the signaling was initiated by either TSHR or IGF-IR. They revealed that the actions of rhTSH, rhIGF-I and GD-IgG could be inhibited, strongly suggesting that the two receptors are functionally linked. Based on those findings, we proffered that IGF-IR might be targeted therapeutically with either monoclonal antibody or small molecule inhibitors of IGF-IR for TAO (10). The capacity for IGF-IR to crosstalk with additional proteins is becoming increasingly recognized (121). Several years after the initial observation of Tsui et al., β-arrestin was found to function as a scaffold for both TSHR and IGF-IR (122), This association potentially accounts for protein:protein crosstalk as underlying the apparent importance of IGF-IR activity in TSHR signaling. Similar associations have been identified in other receptor complexes.

## Culmination of Evidence That IGF-IR Represents A Clinically Important Therapeutic Target in TAO

To test the central hypothesis that IGF-IR represents not only a critical component in the pathogenesis of TAO, but can also be therapeutically targeted, two placebo-controlled, multicenter clinical trials of teprotumumab have been conducted (123, 124). The two studies were designed similarly. The drug was developed as an antineoplastic agent and had already been administered to hundreds of patients with a broad range of cancers (107–111). In general, teprotumumumab (AKA R1507) was well-tolerated in those earlier studies, frequently involving fragile patients; however, the drug was not devoid of adverse events which were more severe in younger patients (125). Like other biologicals targeting IGF-IR simultaneously under development at several other pharmaceutical companies, the efficacy of teprotumumab was found inadequate for sustaining its development program by Roche (107, 109–111, 126). The failure of teprotumumab in the cancer space had made it available for potential repurposing in TAO.

The initial trial of teprotumumab in TAO was organized by River Vision Development starting in 2010. This study, a multicenter phase 2 trial, involved the recruitment of 88 patients within 9 months of developing ocular manifestations of GD (123). The trial enrolled patients with moderate to severe, active TAO between 18 and 75 years of age between July 2, 2013 and September 23, 2015. Patients were clinically euthyroid (within 50% above or below the normative range for serum thyroxine and triiodothyronine levels) at baseline. None had undergone orbital radiotherapy or remedial surgery for TAO. Further, none had received > 1 gram of prednisolone or equivalent for the treatment of TAO. For those with systemic exposure, a uniform steroid washout period of 6 weeks was required prior to study enrolment. Each patient was randomized to receive either placebo or teprotumumab in a 1:1 ratio. Doses were administered as 8 infusions, each at 3 week intervals over a 24-week treatment phase. The initial (partial) dose (10 mg/Kg B.W.) was followed by doses of 20 mg/Kg B.W. The primary response endpoint was the aggregate of 1) ≥ 2-point improvement in clinical activity score (CAS) using a 7-point scale AND 2) ≥ 2 mm proptosis reduction. Both responses must have occurred in the more severely affected (study) eye assessed at 24 weeks. This response must have occurred in the absence of a similar worsening in the contralateral (fellow) eye. Secondary responses included reduction from baseline in proptosis ≥ 2 mm, improvement from baseline in CAS ≥ 2 points, (both measured as continuous independent variables), improved subjective diplopia, and improved quality of life using a validated instrument (GO-QOL) (127). The results of the study were as follows: Twenty-nine of 42 patients in the intention to treat cohort receiving teprotumumab achieved the primary response at 24 weeks compared to 9/45 individuals in the placebo group (p<0.001). The differences between the two treatment groups emerged at week 6 of treatment (p<0.001). These highly significant differences continued over the duration of the treatment phase (p<0.001 at all clinical assessments). The time to first response was significantly shorter in those patients receiving teprotumumab. Further, more subjects receiving the active drug achieved a “high” primary response (≥3 mm proptosis reduction AND CAS reduction ≥ 3 points, p<0.001). With regard to the secondary endpoints, reduction in CAS and proptosis from baseline was significantly different in the two treatment groups as was improvement in the visual function scale of GO-QOL. Subjective diplopia response rates were significantly higher in those receiving teprotumumab compared with the placebo group. The drug safety profile from that trial was encouraging (123). The most common adverse events were muscle cramping and hyperglycemia, most commonly seen in patients with baseline abnormalities in glycemic control or frank diabetes mellitus. The worsening of glycemic control was easily managed with adjustment of diabetes medication. Further, these changes reverted to baseline following completion of the treatment phase of the trial. At analysis it was discovered that an imbalance of smokers occurred with more representation of tobacco users in the placebo group.

**Figure 5:**
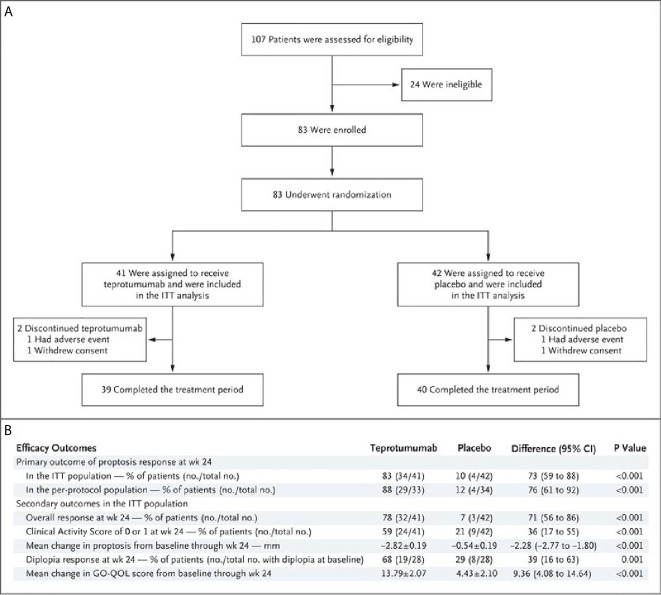
**(A)** Trial profile describing enrollment, randomization, and follow-up. Random assignment to subgroups receiving intravenous infusions of either teprotumumab (10 mg/Kg B.W. for test infusion and 20 mg/Kg for subsequent infusions) or placebo. Infusions administered every 3 weeks for 21 weeks eight infusions in total. **(B)** Efficacy Endpoints describing the phase 3 trial of teprotumumab in patients with moderate to severe active TAO. CMH weighting was used to estimate the common risk difference and the 95% Confidence Interval (95% CI) of the common risk difference for the primary and secondary endpoints of overall responder, percent with CAS 0 or 1, and diplopia responder; least squares mean difference was calculated for secondary endpoints of change in proptosis from baseline and change in Graves’ Orbitopathy quality of life (GO-QOL) questionnaire from baseline using the Mixed-Model Repeated-Measures (MMRM) analysis of covariance (ANCOVA). From N. Engl. J. Med, Douglas R.S, Kahaly G.J., Patel A., Sile E.H.Z., Thompson R. et al. Teprotumumab for the treatment of active thyroid eye disease. 382; 341-352. Copyright ^©^ (2020) Massachusetts Medical Society.

**Figure 6:**
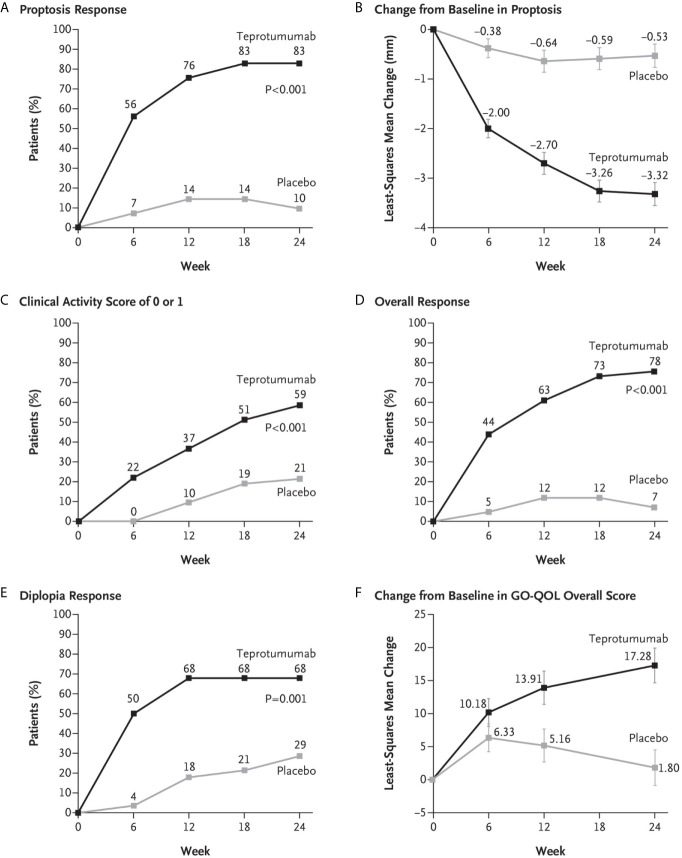
**(A)** Proptosis responder analysis (percent of patients with ≥2 mm reduction in proptosis from baseline in study eye). **(B)** Change from baseline in proptosis (least squares mean ± standard error). **(C)** Percent of patients with clinical activity score (CAS) of 0 or 1 in study eye. **(D)** Overall responder rate (percent of patients with ≥2-point reduction in CAS and ≥2 mm reduction in proptosis from baseline in study eye). **(E)** Diplopia response (percent of patients with improvement of at least 1 grade from baseline). **(F)** Change from baseline in transformed GO-QOL score (least squares mean ± standard error). From N. Engl. J. Med, Douglas R.S, Kahaly G.J., Patel A., Sile E.H.Z., Thompson R. et al. Teprotumumab for the treatment of active thyroid eye disease. 382; 341-352. Copyright ^©^ (2020) Massachusetts Medical Society. Reprinted with permission.

**Figure 7:**
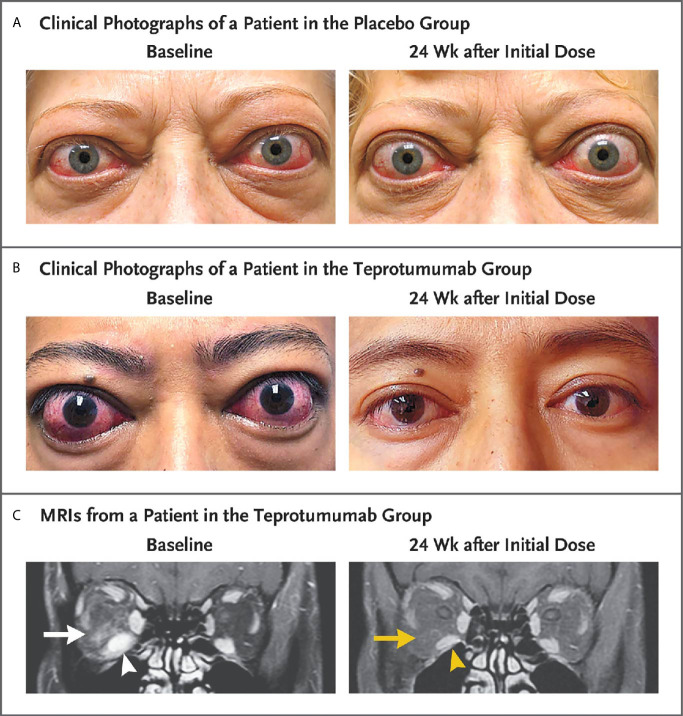
Facial photographic images and MRIs at Baseline and 24 Weeks following treatment with either placebo or teprotumumab in patients enrolled in the Phase 3 trial. Panel **(A)** Clinical photographs of a patient receiving placebo. At baseline, the patient exhibits substantial proptosis (left eye, 29 mm and right eye, 27 mm) as well as multiple inflammatory signs (left eye Clinical Activity Score of 7 and right eye 5). At week 24, considerable proptosis and inflammatory signs remain. **(B)** Images of a teprotumumab-treated patient. Baseline proptosis (both eyes 24 mm), edema, upper and lower eyelid retraction, and multiple inflammatory signs (CAS 5 bilaterally). At week 24, considerable bilateral reductions in proptosis (−5 mm) and CAS (−4 points). **(C)** Coronal, contrast-enhanced, fat-saturated, T1-weighted MRIs in a single patient receiving teprotumumab at baseline and at week 24. Note marked enhancement of the inferior rectus muscle (white arrowhead) and orbital fat (white arrow) as well as inferior rectus muscle enlargement. At week 24, resolved inferior rectus muscle (yellow arrowhead) enhancement and orbital fat (yellow arrow). The muscle volume was reduced by 49% (yellow arrowhead). Proptosis reduction decreased from 23 mm at baseline to 18 mm at week 24. From N. Engl. J. Med, Douglas R.S, Kahaly G.J., Patel A., Sile E.H.Z., Thompson R. et al. Teprotumumab for the treatment of active thyroid eye disease. 382; 341-352. Copyright ^©^ (2020) Massachusetts Medical Society. Reprinted with permission.

A second, phase 3 trial, was initiated by Horizon Pharmaceuticals (now Horizon Therapeutics) after a licensing agreement was established with River Vision in 2017. Eighty-three patients with moderate to severe TAO who had disease and demographic characteristics very similar to those included in the phase 2 trial were enrolled at performance sites in North America and Europe. This occurred from October 24, 2017 until August 31, 2018 (124) (**Figure 5**). The participating investigators represented a subset of those enrolling patients in the phase 2 trial. Subjects aged 18-80 years, were randomized to receive either teprotumumab (n=41) or placebo (n=42). Like the initial study, this trial was also placebo-controlled, double-masked, and all patients were clinically euthyroid, and were judged to manifest active, moderate to severe TAO. All had eye disease ≤ 9 months in duration. Individuals who had previously undergone orbital surgery, had received tocilizumab or rituximab or who had been treated with high-dose glucocorticoids for TAO (excepting those receiving < 1 gm prednisone equivalent following a 6-week washout period) were excluded. The primary outcome was changed from that in the initial study to a reduction in proptosis ≥ 2 mm in the study eye at week 24. The aggregate of ≥ 2 mm reduction in proptosis and improved CAS ≥ 2 points (the overall response and the primary response in the phase 2 trial) was among the secondary end points. Others included improved CAS > 2 points, reduction in proptosis, both measured as independent variables from baseline, reduced diplopia ≥ 1 Gorman scale grade and mean change in the GO-QOL questionnaire score. Trial results of the phase 3 study were congruent with those observed in phase 2. In addition, the skewed distribution of smokers in the two treatment groups in the earlier trial was successfully corrected. More patients receiving active drug experienced a ≥ 2mm proptosis reduction at week 24 when compared to those receiving placebo (teprotumumab 83% versus placebo controls 10%, p<0.001) (**Figure 6**). The necessary number to treat was 1.36. Further, all secondary endpoints were more frequently achieved in patients receiving teprotumumab than those in the placebo group. A few patients underwent orbital imaging at a single performance site at baseline and again at week 24. Those studies were conducted off protocol and revealed that both orbital fat and extraocular muscle volumes were reduced in 6 patients undergoing imaging (**Figure 7**). The phase 3 trial included an extension where all non-responders were offered teprotumumab as an open label, regardless of whether or not they had received the active drug or placebo during the 24-week treatment phase. A similar fraction of patients responded to the drug as did those in the initial intervention phase. Follow-up data, including those from the extension study of this phase 3 trial, reveal that a majority of both proptosis and diplopia responders at Week 24 maintained their responses (56% and 58%, respectively). The aggregate results from the two trials indicate that clinical improvement of moderate to severe, active TAO is very similar to the best outcomes of the ophthalmic remedial surgeries thus far reported in the literature.

Aggregate safety data from the two trials suggest that teprotumumab was well-tolerated. Several adverse events, most mild to moderate in severity, were identified. Among the most common was hyperglycemia, especially in individuals who were diabetic or glucose intolerant at baseline. Grade 2-3 hyperglycemia developed in some patients with pre-study diabetes mellitus who were receiving teprotumumab. These were managed by increasing diabetes medications. No ketoacidosis occurred in this group of patients. Baseline diabetes medication requirements returned to pre-study levels following the completion of the 24-week treatment phase. A few patients in both treatment arms not having histories of carbohydrate intolerance developed grade 1 hyperglycemia. Other adverse events include hearing abnormalities, muscle cramps, hair loss, dysgeusia, and diarrhea. These uniformly resolved or improved substantially after the treatment phase of the trials had been completed.

## Teprotumumab Becomes First Ever FDA-Approved Medical Therapy for TAO

Based on the results of the two clinical trials conducted for teprotumumab in TAO, the FDA approved its use in that disease in January, 2020 (128). Several important exclusions in the profiles of eligible patients who were enrolled in those studies have resulted in additional questions needing answers during the post-approval period. For instance, all trial subjects must have manifested TAO for ≤ 9 months prior to their study enrolment, leaving uncertain whether more chronic, less active disease might respond to teprotumumab. Enrollment criteria were stringently skewed toward early disease since *a priori* reasoning suggested that the most active patients were more likely to respond. The relatively short term follow-up of the studies has left the question of long-term therapeutic durability of the drug. The efficacy of the drug in stable disease is being addressed, not only in the formal study currently under development but also by monitoring real-world experience. Potential effectiveness of teprotumumab in vision threatening TAO resulting from compressive optic neuropathy is unknown since patients with signs of impending vision loss were excluded from both trials. Those open questions are also under study. Single case reports are now appearing suggesting that the drug may be beneficial in apparently stable, longer term disease (129) and in optic neuropathy (130, 131). Clearly more extensive clinical experience with the drug will be necessary before teprotumumab can be considered a reliable treatment option in long-standing or vision-threatening TAO. An important goal of this medical therapy is to lessen reliance on either routine surgical rehabilitation for chronic disease or urgent surgical intervention in sight-threatening TAO.

## Are Disease Indications Beyond TAO in Store for Teprotumumab?

The IGF-I pathway regulates a vast array of physiological and pathological processes in most mammalian tissues. The effective and well-tolerated treatment with teprotumumab of TAO suggests that this pathway might be beneficially targeted in many other diseases. With regard to GD, pretibial myxedema, a potentially debilitating and disfiguring condition seen in a subset of those with TAO, might also improve with the drug. In fact, a recent case report from Varma et al. suggests that long-standing pretibial myxedema might also respond to teprotumumab (132). Shortly after the original observations concerning anti-IGF-IR antibody involvement in TAO (9, 115), similar findings were reported in rheumatoid arthritis (RA) (133). That study demonstrated that synovial fibroblasts from patients with RA responded to their own IgGs as well as to GD-IgG in inducing IL-16 and RANTES expression. Thus, it remains possible that teprotumumab and other IGF-IR inhibitors might prove effective in in the treatment of RA and allied autoimmune diseases. Investigators have speculated that teprotumumab might prove effective in arresting the deleterious lung tissue remodeling associated with coronavirus-19 (134). It remains possible that additional indications for the therapeutic targeting of IGF-I and its pathway will become identified as the experience with teprotumumab broadens.

## Author Contributions

The author confirms being the sole contributor of this work and has approved it for publication.

## Conflict of Interest

The author declares US Patents covering the use of IGF-1 receptor inhibitors in autoimmune disease and consultancy for Horizon Therapeutics Consultant Immunovant.
